# Thermo-Mechanical Treatment for Reducing the Wear Rate of CuCrZr Tool Electrodes during Electro-Discharge Machining

**DOI:** 10.3390/ma16206787

**Published:** 2023-10-20

**Authors:** Jacek Skiba, Mariusz Kulczyk, Sylwia Przybysz-Gloc, Monika Skorupska, Julita Smalc-Koziorowska, Mariusz Kobus, Kamil Nowak

**Affiliations:** 1 Institute of High Pressure Physics, Polish Academy of Sciences (Unipress), Sokołowska 29/37, 01-142 Warsaw, Poland; mariusz@unipress.waw.pl (M.K.); sylwia@unipress.waw.pl (S.P.-G.); monikaw@unipress.waw.pl (M.S.); julita@unipress.waw.pl (J.S.-K.); 2 GEMET, Lisia 16B, 05-410 Józefów, Poland; m.kobus@obrobkametalicnc.pl (M.K.); k.nowak@obrobkametalicnc.pl (K.N.)

**Keywords:** hydrostatic extrusion, electro-discharge machining (EDM), microstructure refinement, electrical discharge wear

## Abstract

The research presented in this paper focused on optimising the process of unconventional plastic forming by hydrostatic extrusion (HE) with post-processing heat treatment of a copper alloy (CuCrZr) for electro-discharge machining (EDM) applications. The treatment was carried out in such a way as to obtain a material with an improved microstructure, characterised by a significant increase in hardness and strength while maintaining a high electrical conductivity, thus achieving the main goal of reducing electrode wear in the EDM process. As part of the research, a material with an ultrafine-grained structure was obtained with an average grain size of *d*_2_ = 320 nm and a much higher strength of *UTS* = 645 MPa compared to the material in the initial state (*UTS* = 413 MPa). The post-processing treatment (ageing) allowed us to obtain a material with a high electrical conductivity after the HE process, at 78% IACS. The electrodes made of CuCrZr subjected to HE had a reduced electrical discharge wear in relation to electrodes made of the initial material. The best results were obtained for electrodes made of the material subjected to a five-stage HE process combined with ageing at 480 °C for 1 h. The electrical discharge wear in these electrodes was reduced by more than 50% compared to electrodes made of non-deformed copper.

## 1. Introduction

Electro-discharge machining (EDM) is an unconventional machining process, providing an alternative to standard and common machining-based processes. The first reports describing the use of EDM date back to around 1943 [1]. The basis of the aforementioned EDM is the phenomenon of a violent electrical discharge created in the contact zone between the working electrode and the hollow material. The energy created at the material boundary in the so-called working gap leads to melting of the workpiece material and its vaporisation. The advantage of the aforementioned technology is that its only prerequisite is that the hollow material must conduct electric current; an undoubted advantage and an advantage over other machining methods is that good mechanical properties such as a high hardness and strength are not an obstacle. This means that EDM can be successfully used for the machining of difficult-to-cut metallic materials, including composites. In addition, due to the lack of the conventional cutting forces and stresses associated with them, the method enables the production of holes with very small diameters of up to several micrometres, which cannot be done using conventional machining methods [2,3,4,5]. The specificity of the EDM process means that the key properties of materials for electrodes are their thermophysical properties, such as conductivity, thermal expansion, melting point and electrical conductivity [6]. Although the EDM process does not involve mechanical forces like in conventional machining, the sparks generated act violently and generate mictrostresses in the machined material [7,8]. Therefore, mechanical and structural properties have a huge impact on both the EDM process and the life of the electrodes. Another important factor when choosing an electrode material is its machinability. Difficult-to-cut materials require complex machining techniques, generating high costs in electrode production, which significantly limit their application in the EDM process [6,9]. Copper is the most commonly used material for EDM electrodes due to its high electrical and thermal conductivity. Despite this, however, it has some disadvantages, such as high thermal expansion and a low melting point. These properties can negatively affect the process of removing “spoil” from the machining zone, thus leading to increased wear of the electrode [10]. Another disadvantage of copper is that it is a ductile material, which makes it difficult to obtain a suitable electrode surface quality through machining [11]. To change this, alloying dopants, such as tellurium, are used, but they have a negative effect on electrode wear [7]. Due to the above disadvantages and limitations, the authors attempted to develop a new material for EDM electrodes based on a CuCrZr copper alloy subjected to plastic working in combination with a post-deformation heat treatment. This is the preferred method of machining the tested CuCrZr alloy due to its susceptibility to hardening in the process of plastic strain and the precipitation hardening generated in the process of ageing by the Cr and Cu-Zr phases [12,13,14,15]. This type of research project has been widely conducted in recent years, but has mainly been limited to Equal Channel Angular Pressing (ECAP), and the primary objective was to achieve the maximum strength properties, and not to optimise them, in relation to application in the EDM process [14,16,17]. The products obtained in multi-stage ECAP cycles were subjected to a thermal precipitation treatment to trigger the process of ageing before or after the ECAP process [17,18,19], which led to a significant increase in strength while maintaining a high electrical conductivity. In the specialist literature, one can also find research projects involving unusual processes, such as dynamic plastic strain at the temperature of liquid nitrogen (DPD-LNT) without ageing [13]. The process allowed us to obtain a material with a nanocrystalline structure with a strength of *UTS* = 700 MPa and an electrical conductivity of 78.5% *IACS*. In turn, the combination of the DPD-LNT technique with an additional post-deformation heat treatment (ageing) allowed us to obtain even a higher strength parameter of UTS = 832 MPa, with a simultaneous decrease in electrical conductivity to 71.2% *IACS* [15]. The authors of the present study attempted to apply an unconventional method of high-pressure plastic forming in the form of hydrostatic extrusion (HE), which made it possible to improve the functional properties of the investigated CuCrZr copper alloy, which has application potential in the EDM process as an electrode material. Hydrostatic extrusion, thanks to the prevailing triaxial compressive–tensile stresses in the deformation zone, allows efficient deformation of materials with high degrees of deformation while maintaining their homogeneity both microstructurally and mechanically. It is believed that significant microstructure refinement combined with a well-developed grain texture will enable a good compromise between the strength and electrical conductivity of the CuCrZr alloy, which will consequently allow its application as an electrode in the EDM process. Research conducted at the Institute of High Pressure Physics, Polish Academy of Sciences (IHPP PAS), over 45 years has confirmed the exceptional suitability of HE to generate severe plastic deformation in materials. It has been proven repeatedly that hydrostatic extrusion promotes grain refinement with a higher efficiency than other SPD methods, also due to the higher strain rates used [20,21,22,23] and the favourable state of compressive and tensile stresses present in the deformation zone. This confers the materials with a better strength [24,25,26,27], fatigue properties [28], impact strength [29], tribological properties [30] and corrosion properties [31], while improving functional properties such as the machinability [32,33] and electrical conductivity [34].

In the present study, the authors attempted to optimise the combination of two methods of strengthening, i.e., plastic deformation using the HE method and heat treatment in the form of aging. The structure of the studied CuCrZr alloy, obtained as a result of the synergistic combination of the two methods, led to the modification of material properties such as strength, yield strength and electrical conductivity.

Many publications on the modification of the microstructure and properties of materials after severe plastic deformation indicate its potential application. However, ongoing research is limited to purely scientific studies without considering technological considerations. In an era of intensive development of unconventional techniques for manufacturing and modifying materials, it is necessary to analyse their performance properties in real conditions concerning their operation in detail. The HE process offers the possibility of the actual use of plastic-machined materials in industry. Application studies involving wear analysis under machining conditions confirm this fact and undoubtedly constitute an element of innovation in the present publication in relation to studies available in the literature.

The results of the application studies presented in this paper allow the market implementation of a new unique product in the form of electrodes for the EDM process of ultrafine-grained CuCrZr alloyed copper.

## 2. Materials and Methods

A CuCrZ copper alloy with the chemical composition shown in Table 1 below was tested.

Before the process of hydraulic extrusion, the material was subjected to solution heat treatment at 1000 °C for 1 h and cooled in water. The properties of the tested CuCrZr alloy in the initial state and after the solution heat treatment are listed below in Table 2.

The material was subjected to the process of plastic deformation by hydrostatic extrusion in two processes:

(a) One-stage extrusion—including deformation with three different degrees of strain ranging from *ɛ* = 1.12 to 2.53.

(b) Cumulative extrusion—involving multiple deformations of the material with increasingly smaller degrees of strain with the cumulative strain after five stages of HE equalling *ɛ_cum_* = 3.89.

The hydrostatic HE extrusion process was carried out in presses with nominal pressures of up to 1800 MPa. HE stations were equipped with a cooling system to effectively minimise the adiabatic heating effect of the extruded product.

**Figure 1:**
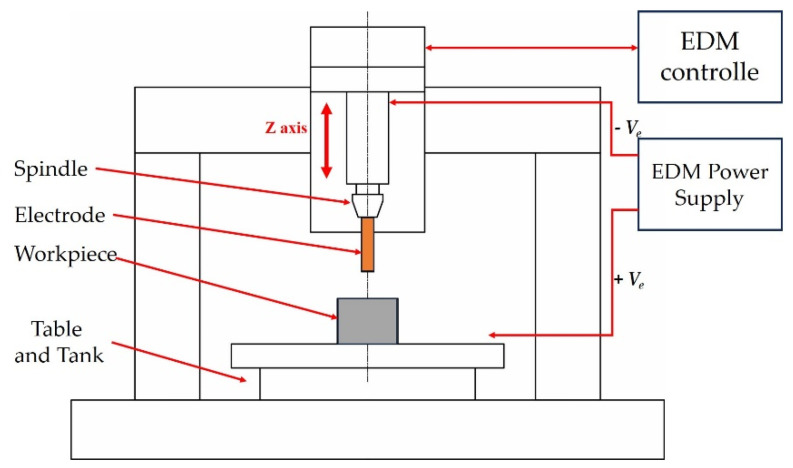
Wear test bench for the EDM process.

Analysis of the microstructure in the initial state was carried out using light microscopy with a Nikon Eclipse LV150 microscope (Tokio, Japan). Microstructures after the HE process were determined using transmission electron microscopy with a TEM JEOL 1200 EX microscope (Croissy-sur-Seine, France). Observations were made of both cross-sectional and longitudinal sections. The quantities were estimated using Micrometer V-1.0 software [35]. Analyses were performed based on TEM images, where the equivalent diameter d2 was calculated after imaging and mapping at least 200 grains randomly selected from the population. Mechanical property tests were conducted using a Zwick-Roell Z250 static testing machine (Ulm, Germany) with a maximum force of 250 kN. Based on the stress/strain curves, the tensile strength, UTS, yield strength, YS, and elongation to rupture, A, were determined. The tests were conducted at a tensile rate of 0.008 s-1 on five specimens of 6 mm diameter taken along the axis of the bars. Microhardness measurements were made on the cross-section of the extruded bars using a Zwick-Roell ZHV1-A hardness tester (Ulm, Germany) under a load of 200 g for 15 s. Tests of electrical conductivity (%IACS) were conducted on the longitudinal and transverse sections of the tested materials using a SIGMATEST 2.069-Forester device (Frontier, Singapore). Tests were carried out on the cross-section and the longitudinal section of the copper samples after the HE process. Electroerosion wear tests were carried out on an EDM process laboratory station equipped with a conventional analogue EDM machine EDMA-40, Figure 1. EDM test specimens were made in the form of cylinders with a diameter of 10 mm and a length of 65 mm. The specimens were made by machining (turning) with the following parameters: cutting speed, *Vc* = 600 rpm; feed rate, *f* = 0.1 mm/rev.

The machining process was performed with intensive cooling to prevent intense heating of the material and degradation of the fragmented microstructure in the HE process.

Wear tests were conducted based on the maximum and minimum capabilities of the EDM apparatus and are shown in Table 3. Parameters were established based on the VDI 3400 standard set by Verein Deutscher Ingenieure (VDI), the Association of German Engineers [36]. This is a standard used mainly in the EDM industry, but nevertheless functions universally. The maximum EDM parameters chosen for the tests (*I_r_* = 12 A, *t* = 9 ms) allow hollowing according to VDI standard 3400#40, which corresponds to a surface roughness equal to *Ra* = 10 µm, i.e., rough machining. The minimum EDM parameters (*I_r_* = 3 A, *t* = 4 ms) allow hollowing according to the VDI 3400#22 standard, which corresponds to a surface roughness equal to *Ra* = 1.25 µm, i.e., finish machining.

## 3. Results and Discussion

### 3.1. Hydrostatic Extrusion

The parameters of the HE process are given in Table 4 below.

The recorded characteristics of pressure as a function of time for individual HE processes, both single-stage and cumulative, confirmed the correctness of the adopted technological parameters, such as the die angle, the deformation speed and the method and type of lubrication.

**Figure 2:**
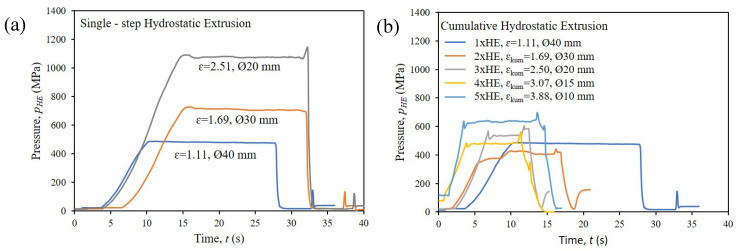
Pressure characteristic of hydrostatic extrusion of the CuCrZr copper alloy: (**a**) single-step HE, (**b**) cumulative HE.

The characteristics presented in Figure 2 show a stable, almost linear tendency with a clear flattening corresponding to the extrusion pressure, *p_HE_* (MPa). The value of the extrusion pressure is strictly dependent on the mechanical properties of the material and its susceptibility to plastic deformation. Despite the process of hydraulic extrusion being carried out under intensive cooling of the extruded product, the adiabatic temperature of the process for individual stages of the test was estimated. This aspect is particularly important for the materials susceptible to thermally induced healing and recrystallisation processes, the group of materials that the CuCrZr copper alloy belongs to. The effect of strong adiabatic heating has been analysed multiple times in works describing the HE process [21,22,37]. It is the direct result of the mechanical work generated during deformation that is converted into heat [37,38]. The value of the adiabatic temperature of the process of hydraulic extrusion can be estimated using the following formula:

Δ*T = β∙p_HE_*/(*ρ∙cp*) (1)

where:

Δ*T*—adiabatic temperature;
*ρ*—material density;
*cp*—specific heat;
*p_HE_*—extrusion pressure;
*β*—dimensionless parameter denoting the internal plastic work converted into heat during deformation.

Therefore, assuming the density of the tested CuCrZr alloy was ρ = 8.9 g/cm^3^, the specific heat *cp* = 0.380 Jg^−1^K^−1^ [39] and the parameter β was 0.9, which results from the specific conditions prevailing during the hydraulic extrusion conducted with a high strain rate. A significant increase in temperature was observed. For one-stage processes, the recorded temperature increase was from 127 to 285 °C, i.e., from 0.29 to 0.41 of the homologous temperature, Th (defined as the ratio of the calculated temperature to the melting point *T_h_* = *T/T_m_* in Kelvin), while for the cumulative process, the recorded temperature increase was from 119 to 169 °C, i.e., from 0.28 to 0.33 Th. In pure or low-alloyed metals, recrystallisation usually occurs at a temperature of 0.3 *T_m_* [37]. This means that dynamic recrystallisation processes can occur, especially in single-stage HE processes, where the Th values exceed 0.4. On the other hand, in the case of multi-stage extrusion, where the homologous temperature is much lower, one can expect a much more effective strain strengthening of the material resulting from the accumulation of structural defects.

### 3.2. Microstructure Evaluation

**Figure 3:**
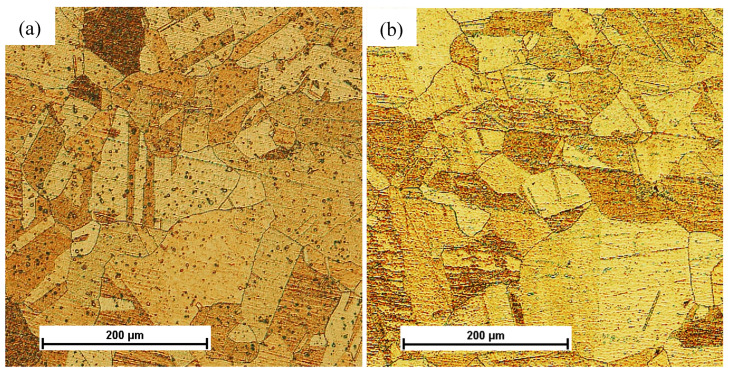
Microstructure of the CuCrZr copper alloy after solution treatment and before deformation: (**a**) cross-section, (**b**) longitudinal section.

The tested CuCrZr alloy in the initial state was subjected to a solution heat treatment at 1000 °C for 1 h, which allowed for a significant increase in plasticity, enabling further shaping using the method of severe plastic deformation (SPD) in the hydrostatic extrusion process. The CuCrZr alloy after the solution heat treatment had a homogeneous isotropic structure with equiaxed grains with an average size of ~80 µm, Figure 3.

A direct factor affecting the final properties of the materials subjected to SPD processes is the microstructure refinement and the introduction of a large number of structural defects, leading to a drastic reduction in thermophysical properties, such as the thermal and electrical conductivity, which are crucial from the standpoint of application in EDM machining. The above has been confirmed by numerous publications showing that an increase in dislocation density in severely deformed CuCrZr alloys is the main cause of decreases in electrical conductivity. These defects become an effective barrier, blocking the flow of free electrons and photons [40]. Therefore, to optimise the properties of the CuCrZr alloy for EDM applications, the effect of post-deformation heat treatment (ageing) on the evolution of the microstructure was investigated. Based on the literature data, ageing after a single extrusion process at a temperature of 480 °C for 1 h was selected for the CuCrZr alloy [34].

**Figure 4:**
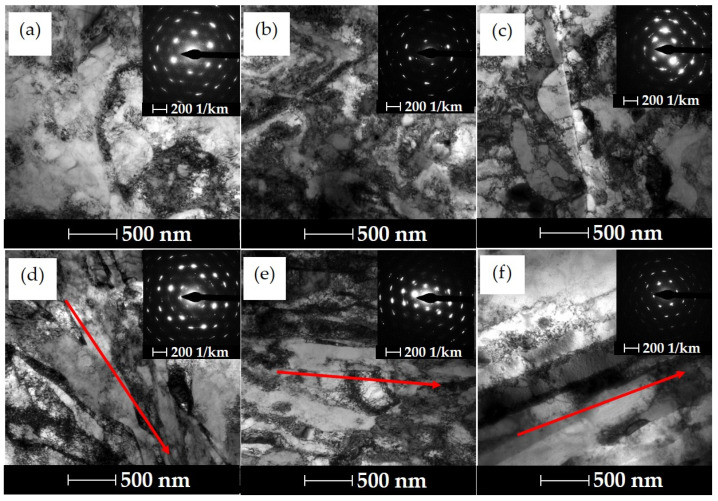
Microstructural changes in CuCrZr alloy cross-sections (**a**–**c**) and longitudinal sections (**d**–**f**) as a function of true strain after aging, for *ε* = 1.11 (**a**,**d**), *ε* = 1.69 (**b**,**e**) and *ε* = 2.51 (**c**,**f**), respectively. The arrow indicates the direction of extrusion.

Figure 4 shows the evolution of the microstructure after a single extrusion. At the lowest degree of strain, *ε* = 1.11, defective primary grains are observed, Figure 4a, and in the longitudinal section, the formation of banding in the microstructure is observed, which is characteristic of the hydrostatic extrusion process, Figure 4d. Strong anisotropy of the microstructure after hydrostatic extrusion was previously observed in other materials, such as aluminium alloys 6060, Armco iron or CuCrZr alloy [34,41,42]. An increase in strain during single extrusion and ageing to the level of ε =1.69 causes an increase in the density of structural defects and locally visible cellular dislocation structures and subgrains, as evidenced by slightly blurred diffraction reflections, Figure 4b. In the longitudinal section, an increased effect of microstructural anisotropy is observed, with a stronger orientation of the microstructure, as well as locally occurring subgrains with fewer internal defects, which may indicate that dynamic healing processes have occurred, Figure 4e. It is worth noting that at the true degree of strain presented in the previous section, an increase in the homologous temperature to the level of *T/T_m_*~0.35 is observed, which significantly favours the occurrence of thermally induced phenomena that weaken the effects of strain hardening. Visible effects of the influence of the released heat on microstructural changes are observed at the largest unit strain, *ε* = 2.51—the temperature exceeded ~ 0.4. In the cross-section, a clearly developed sub-grain structure is observed in the area of larger grains with cleaned internal defects and fuzzy diffraction reflections, Figure 4c, which may indicate the occurrence of dynamic recrystallisation processes in the strain zone during the HE process. In the longitudinal section, an even stronger anisotropy of the microstructure and a similar effect of a lower density of defects inside the emerging bands are observed, Figure 4f.

**Figure 5:**
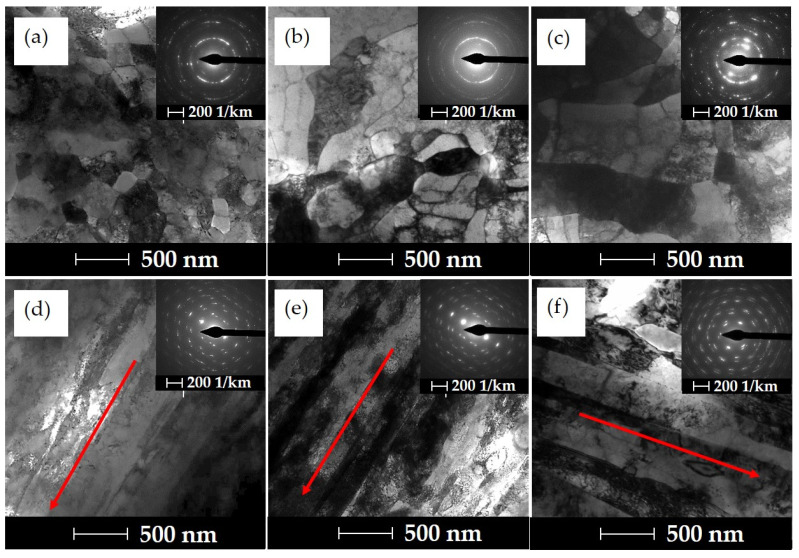
Microstructural changes in the CuCrZr alloy in cross-sectional (**a**–**c**) and longitudinal (**d**–**f**) sections after a cumulative HE process with strain ε = 3.88 and after hourly aging process at *T* = 450 °C (**a**,**d**), *T* = 480 °C (**b**,**e**) and *T* = 510 °C (**c**,**f**), respectively. The arrow indicates the direction of extrusion.

Figure 5 shows the microstructure of the CuCrZr alloy after the process of cumulative hydrostatic extrusion with the total true strain of *ε* = 3.88. The cumulative process makes it possible to increase the total true strain, while limiting the thermal effects during the plastic deformation as an effect of the reduction in successive unit deformations. Due to the very high energy in the form of accumulated defects in the material after such severe strain, ageing processes were carried out at three different temperature levels, i.e., 450 °C, 480 °C and 510 °C, for one hour. The energy accumulated in the material can significantly affect the kinetics of ageing and microstructural changes occurring in a highly deformed material depending on the temperature used. This is already observed at a temperature of 450 °C, where a significant microstructure refinement with locally present clean grains due to healing processes is observed in the cross-section. The presence of grains is indicated by clearly formed rings in the diffraction pattern, Figure 5a. In the longitudinal section, a very strong anisotropy of the microstructure with a strong orientation in the direction of extrusion can be observed, Figure 5d. The average grain size is *d_2_*~210 nm, and the coefficient of variation of its distribution is *cvd_2_* = 0.24. Increasing the temperature of the ageing process to the value of *T* = 480 °C leads to the intensification of thermal processes, and as a result of which, an even more developed grain microstructure is formed with fewer defects, as evidenced by the nature of the diffraction pattern, Figure 5b. The stronger effect of the thermal healing processes causes the average grain size to increase to *d_2_*~320 nm and causes homogenisation of their size, as evidenced by the lower value of the grain size distribution variation coefficient *cvd_2_* = 0.21. The increase in the average grain size is associated with the disappearance of subgrains and the migration of defects into the grain boundary areas. Strong anisotropy of the microstructure in the longitudinal section is still observed, Figure 5e. Increasing the temperature of the ageing process to 510 °C translates into a clear grain growth, Figure 5c. The average grain size is *d_2_*~420 nm and a sharp increase in the grain size distribution variation coefficient to the value *cvd_2_* = 0.45 indicates the occurrence of dynamic recrystallisation processes, leading to a heterogeneous microstructure and weakening the effects of strain hardening. In the longitudinal section, we further observe the effects of microstructure anisotropy in the form of elongated bands, although they are characterised by a significant increase in width, Figure 5f. Recrystallised grains are also observed locally in the longitudinal section.

### 3.3. Mechanical Properties

**Figure 6:**
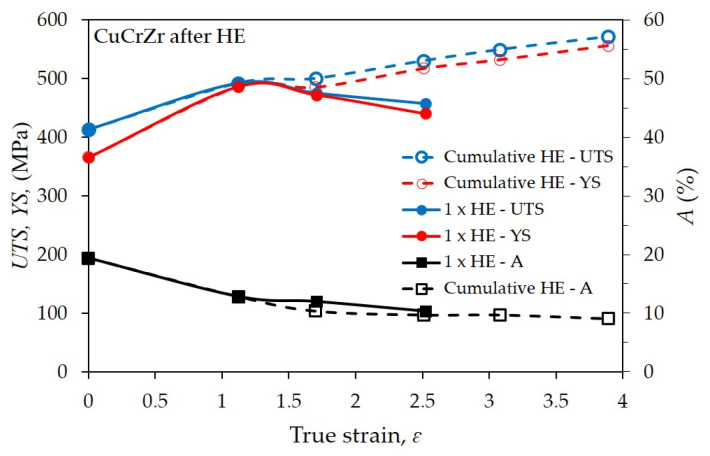
Dependence of tensile strength, *UTS*, and yield stress, *YS*, on true strain, ε, for the CuCrZr copper alloy after single-step cold hydrostatic extrusions and after the cumulative HE.

Figure 6 shows the strength properties of the tested CuCrZr alloy after hydrostatic extrusion as a function of the true strain. The results obtained in the static tensile test reflect the observed structural changes resulting from the applied deformation model—single-stage or cumulative deformation. In single-stage extrusion with increasing unit strain in the range from ɛ = 1.12 to about ɛ = 2.53, the significant influence of adiabatic effects with increasing strain is demonstrated. These effects are due to the low SFE of ~55 mJ/m^2^, which makes Cu and its alloys highly susceptible to recrystallisation and other thermally induced processes, significantly limiting their strengthening efficiency [43,44]. This phenomenon is also confirmed by the estimated values of the homologous temperature, which for the once-extruded samples with deformations of *ɛ* = 1.69 and 2.51 were, respectively, *T_h_* = 0.34 and 0.41 *T_m_*. In pure or low-alloyed metals, recrystallisation usually occurs at a temperature of 0.3 *T_m_*. Therefore, the highest strength was obtained for the first HE stage with strain ɛ = 1.11, and the results obtained were *UTS* = 492 MPa and *YS* = 486 MPa. These values are higher compared to the material in the initial state by about 20% for *UTS* and by more than 30% for *YS*. In the further stages of a single HE, decreases in the value of strength properties were observed. Further strengthening of the material was possible through the use of a cumulative process consisting of extruding the material in several stages with smaller unit deformations. For this purpose, the tested CuCrZr alloy was deformed in a five-stage hydrostatic extrusion process with a cumulative true strain of *ɛ* = 3.89. As a result, further, almost linear increases in *UTS* and *YS* were achieved, with the maximum values for the product with a diameter of Ø10 mm. After five stages of hydrostatic extrusion, the following values were obtained for the tested CuCrZr alloy: *UTS* = 571 MPa and *YS* = 556 MPa. The values achieved, as in the case of the single-stage HE, result from the adiabatic temperature generated during the process, which determines the occurrence of thermally induced healing and recrystallisation processes. In the case of cumulative HE, the homologous temperature in each stage oscillates around 0.3 *T_m_*, which proves a significant reduction in these processes.

**Figure 7:**
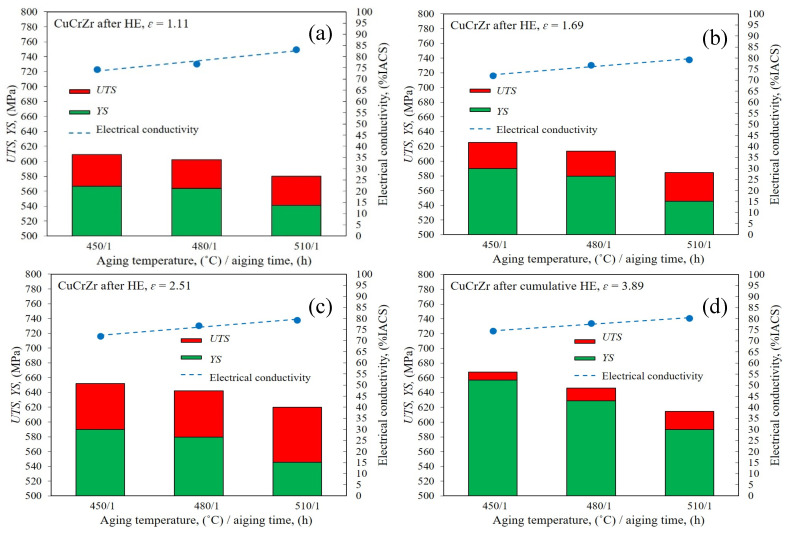
Dependence of *UTS* and *YS* on aging time/aging temperature for the CuCrZr copper alloy after (**a**–**c**) single step HE, (**d**) cumulative HE.

In order to further strengthen the CuCrZr alloy and improve its functional properties, the material was subjected to optimisation via heat treatment in the form of ageing. The authors subjected the tested CuCrZr alloy to the ageing process at temperatures of 450 °C to 510 °C for 1 h. The results were compiled as a function of temperature and electrical conductivity, Figure 7.

The results obtained indicate a higher efficiency for the cumulative process, where a clear increase in *YS* relative to *UTS* was observed. This effect is related to the so-called “Hall-Petch” phenomenon. The relationship describes the relationship between the average grain size and the yield point. The classical assumption is that the main factor through which refinement of the structure affects the strengthening of the material is the piling up of dislocations at grain boundaries, resulting in an increase in the force required to deform the material according to the Hall–Petch equation [45,46]. The analysis carried out indicates a clear influence of the ageing temperature on the strength of the tested CuCrZr alloy. The highest strength values were obtained for the sample after five HE processes with a cumulative true strain of *ɛ_cum_* = 3.89 and post-deformation ageing at 450 °C, for which *UTS* = 668 MPa and *YS* = 657 MPa, with an electrical conductivity of 74% *IACS*. With the increase in the ageing temperature, a decrease in strength and a simultaneous, almost linear increase in electrical conductivity were observed, Figure 7. It is also worth noting that with the increase in the ageing temperature of the cumulatively extruded sample, Figure 7d, an increase in the difference between *UTS* and *YS* was also observed. This proves the occurrence of healing and recrystallisation in the material, and consequently, degradation of the microstructure and grain growth. The effects of recrystallisation processes at 510 °C, presented in the previous section, clearly reflect the observed changes in mechanical properties. At the lowest tested temperature, i.e., 450 °C, as shown in Figure 7a, these effects do not occur. Moreover, it was observed that the difference between *UTS* and *YS* drops to only 6 Mpa after ageing at 450 °C. This is probably due to the healing processes taking place, where the annihilation and segregation of defects do not have a destructive effect on the refinement of the microstructure. These effects are not observed in samples after the one-stage HE process, Figure 7a–c, for which the differences between *UTS* and *YS* are much larger. This is directly due to the greater efficiency of microstructure size reductions in the cumulative process, which can be seen in the pictures of the microstructure, Figure 5.

Tests involving the combination of deformation and precipitation hardening on the CuCrZr alloy were carried out many times, but they were aimed at achieving maximum values, and occasionally they were combined with application tests to verify their actual operational values. Table 5 below summarises the properties of the tested CuCrZr alloy obtained in this work in comparison with the literature data.

The first two results show the highest strength values for, respectively [15], *UTS* = 832 MPa and [13] *UTS* = 700 MPa. Both results were obtained with the samples after an unconventional and complex process of dynamic plastic deformation at the temperature of liquid nitrogen (LNT-DPD). In addition, the small size of the obtained samples excludes their application in the industry. Also, tests involving cold rolling and post-deformation ageing [48], despite very high values of electrical conductivity equal to 86.8% *IACS*, do not support their use in industry or in high-volume products, especially in relation to the application which is the subject of the present study, i.e., EDM electrodes. This is due to the very small thickness of the products (0.4 mm). In other cases, mainly in tests based on the ECAP method, much lower values of electrical conductivity were obtained of [16] 68.8% *IACS* and [47] 73% *IACS*, respectively. This is due to the microstructure morphology characteristics of the ECAP process. There is a very large number of structural defects, forming an effective barrier for the current flow. Only the research carried out by Feng et al. on cold drawing with post-deformation aging at 450 °C allowed the production of the material in a volume enabling the production of EDM electrodes while maintaining a high electrical conductivity of 78% *IACS* [12]. Although the electrical properties obtained in the drawing process allow for their practical application, the results obtained by the authors in this work, as well as in previous studies [34], confirm the significantly higher efficiency of the process of hydrostatic extrusion. *YS* values obtained through the process of hydrostatic extrusion were more than 100 MPa higher than those obtained in the process of drawing, while an equally high electrical conductivity of 78% *IACS* was retained. An example of an application is shown in the authors’ own research [34]. However, the study covered only the testing of the CuCrZr alloy after a one-stage HE process with a strain of *ɛ* = 2.28. In the course of the research, the authors optimised the thermo-mechanical processing of the CuCrZr alloy to produce electrodes with the high hardness required for the process of resistance welding. The best combination of hardness and electrical conductivity was obtained through ageing at 480 °C for 1 h, where a hardness of 180 *HV10* and electrical conductivity of 79% *IACS* were obtained. As a result of thermo-mechanical processing, including HE and post-deformation ageing, a *UTS* of ~630 MPa and a *YS* of 610 MPa were obtained. The results obtained in this study, as far as achieving an ideal trade-off between strength and electrical conductivity is concerned, have surpassed all other literature data on high-volume materials, enabling their application as EDM electrodes. As a result of the optimisation of the thermoplastic process involving five-stage HE with a true strain of *ɛ* = 3.89 and post-deformation ageing in the temperature range of 450 °C to 510 °C, a *UTS* in the range of 679 to 615 MPa, a *YS* in the range of 676 to 590 MPa and an *IACS* in the range of 71.8 to 80.3% were obtained.

### 3.4. Electrical Conductivity

**Figure 8:**
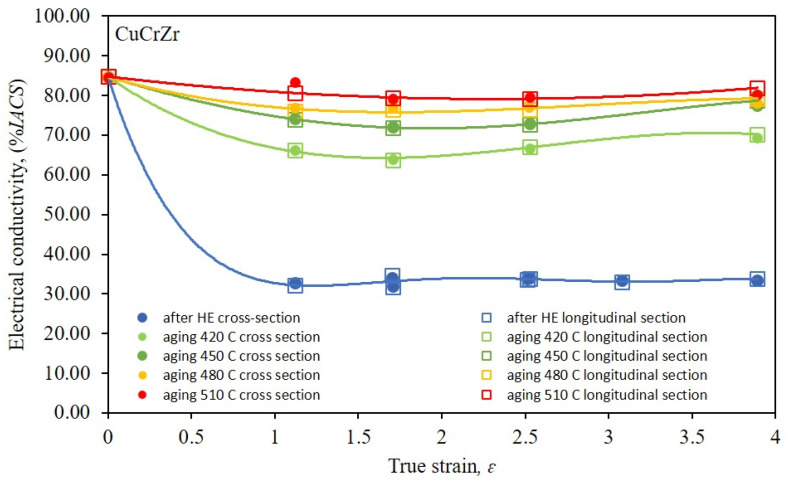
Dependence of electrical conductivity % *IACS* on true strain *ε* for copper after the single-step cold hydrostatic extrusions and after the cumulative HE.

Due to the application potential of the tested copper alloy (CuCrZr) subjected to the process of hydrostatic extrusion, the authors conducted an analysis of the electrical conductivity of samples after all deformation stages, both after one-stage HE and after cumulative HE, Figure 8.

Electrical conductivity measurements were taken at both the cross-section and the longitudinal section of the tested specimens. The results obtained clearly indicate that defects introduced into the material during the process of HE, characterised by a high density of dislocations, led to a decrease in the electrical conductivity, reaching 60% in comparison with the material at the initial state (after solution heat treatment). The decrease in conductivity after the HE process remains at a constant level in the entire range of the tested plastic strain, both in the cross-section and in the longitudinal section of the tested samples. This phenomenon is the result of a much greater efficiency in the formation of defects and the related high density of dislocations in alloy materials, including the tested CuCrZr. This effect was also confirmed in other studies involving SPD processes such as ECAP and described by Liang et al. [47]. The authors subjected the tested CuCrZr alloy to deformation using the ECAP 8x method, which led to a decrease in electrical conductivity to the level of about 30% *IACS*. Therefore, in order to optimise the properties of CuCrZr for commercial applications, it is necessary to apply post-deformation ageing after plastic deformation so as to obtain a structure with the lowest density of defects constituting a barrier to block the flow of free electrons. The results of post-deformation heat treatment tests in the temperature range of 450 °C to 510 °C presented in Figure 8 indicate an increase in electrical conductivity due to the controlled heat-induced processes, indicating healing and recrystallisation took place. However, depending on the temperature used, the share of individual processes varies. At the lowest temperature, a strongly defective microstructure with a large number of structural defects inside the grains appears, which only proves their segregation and polygonisation in relation to the microstructure after HE. With the increase in temperature, the share of the healing and recrystallisation processes increases, reaching a maximum at the highest tested temperature, i.e., 510 °C. As the plastic deformation increases, the unification of the conductivity values for all tested temperatures can be observed. This indicates a greater accumulation of high-energy structural defects that are more prone to undergoing thermally induced processes.

### 3.5. Usability Tests

**Figure 9:**
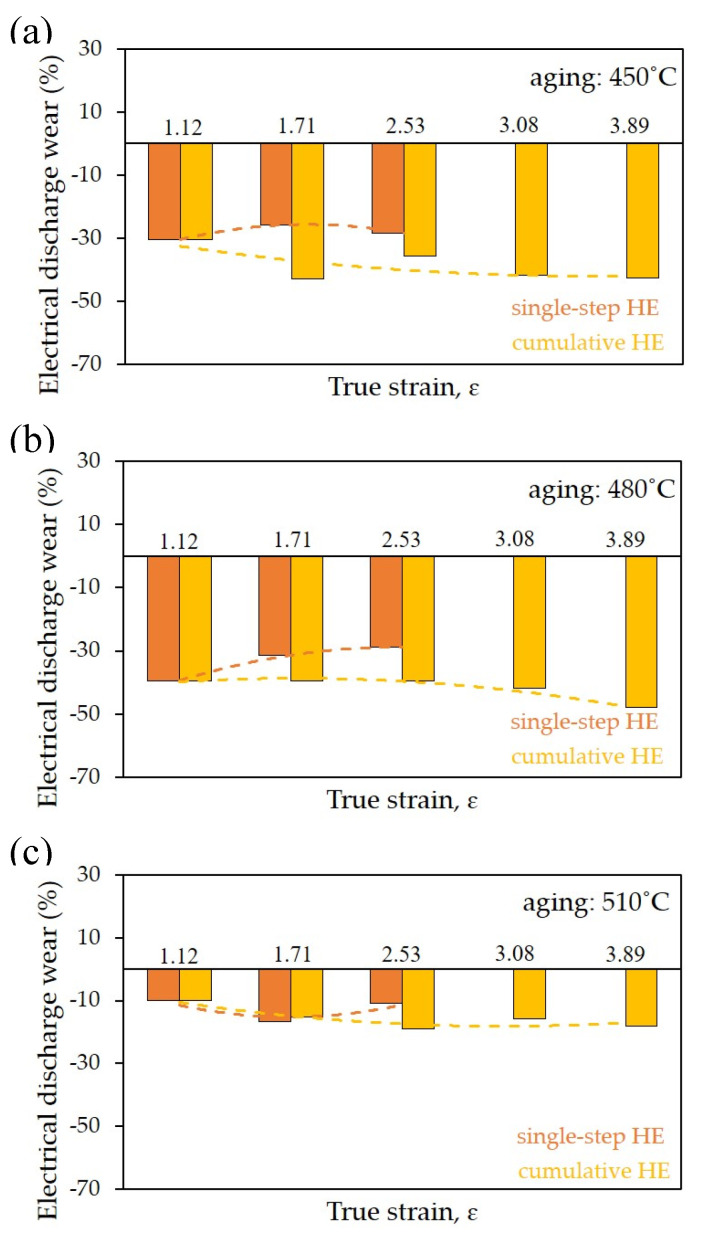
Evolution of electrical discharge wear as the function of true strain *ε* for the CuCrZr electrode after aging in EDM tests: (**a**) 450 °C, (**b**) 480 °C, (**c**) 510 °C.

EDM machining electrodes were made from hydrostatically extruded copper. The electrodes had a diameter of 10 mm and a height of 60 mm. As part of the test, a workpiece made of steel WCL/1.2343/X37CRMOV5-1 was machined. The tests were carried out with two machining processes, i.e., rough machining and finish machining. For reference purposes, electrodes made of non-deformed copper were subjected to similar tests. The results obtained in the wear tests showed a significant advantage of the proposed solution in fine machining. This is because in rough machining, the wear of the electrode is small, while the erosion of the machined material is high. This condition is possible due to the specificity of the process, which consists of the fastest possible rough machining without the need to ensure specific parameters of the machined element, such as a high dimensional tolerance or a low surface roughness. The obtained results of wear after rough machining with electrodes made of the material in the initial state and after HE is about 0.05 mm per 20 mm of machining, and in all measurements the values are within the margin of error. A different situation is observed in fine machining, where the electrode itself is subject to much greater wear. As a result of the greater wear of the electrode, the wear of the machined material is lower. This allows the machine operator to control both the geometry of the EDM-machined products as well as to obtain a precisely defined surface geometry and roughness. The dependence of the degree of electro-erosion wear as a function of actual deformation for electrodes made of the material in the initial state and HE processed materials (both single-stage and cumulative) subjected to the ageing process in the tested temperature range (450 to 510 °C) is presented in Figure 9.

All tested samples has a lower electrical discharge wear than electrodes made of the material in the initial state, marked with a red line on the graph. The percentage wear rate of EDM electrodes after the HE process in relation to those made of the material in the initial state was calculated using the following formula:

(2) $$Edw=\left[1-\frac{{Edw}_{IS}}{{Edw}_{HE}}\right]\cdot 100\%$$

where:

*Edw*—electrical discharge wear;
*Edw_IS_*—electrical discharge wear in the initial state;
*Edw_HE_*—electrical discharge wear after hydrostatic extrusion.

The lowest electrical discharge wear was obtained for electrodes made of the material subjected to the cumulative HE process with a strain of *ɛ* = 3.89 and post-deformation ageing at 480 °C for 1 h, Figure 9b. In this configuration, the wear was lower than the wear observed when using the electrode in the initial state by almost 50%. This effect results directly from the microstructure evolution. The very high applied plastic strain of up to 3.89 led to the formation of a clearly oriented anisotropic microstructure with elongated grains in the direction of extrusion, the boundaries of which were not an effective barrier to the movement of electrons responsible for electrical conductivity. In addition, the post-processing treatment (ageing) contributed to the segregation of structural defects and their annihilation in the grain boundaries, which further improved the flow of electric current. In the case of the one-stage process, an increase in electrical discharge wear with an increase in plastic deformation was observed, Figure 9b. This phenomenon is caused by adiabatic effects (as described above in this paper) in the form of dynamic healing processes and dynamic recrystallisation occurring during the conducted deformation processes. These processes lead to a reduction in the effects of strengthening the material, thus limiting its effectiveness. A similar tendency was observed for the samples subjected to the ageing process at 450 °C, with the EDM wear rate slightly higher in this case, Figure 9a. This is because the temperature of 450 °C used in the treatment did not allow for the annihilation of defects in the grain boundaries, which was also confirmed in electrical conductivity tests (Figure 8) that showed that the samples after the ageing process at 450 °C have a lower electrical conductivity than those treated at 480 °C. On the other hand, in the samples subjected to ageing at 510 °C, a significant increase in electro-erosive wear compared to the samples treated at 450 and 480 °C was observed. This is due to the healing and recrystallisation processes that degrade the fragmented microstructure created in the HE process, which was also confirmed by transmission electron microscopy pictures, Figure 5.

## 4. Conclusions

The presented results for the CuCrZr copper alloy confirmed the effectiveness of the hydrostatic extrusion process as one of the SPD methods used to refine the copper microstructure. As a result of the optimised synergistic combination of a five-step HE process with a culminated true strain of *ɛ* = 3.89 and heat treatment after aging at 480 °C for 1 h, a fine-grained microstructure with a predisposed morphology of elongated grains was obtained, which contributed to achieving the main objective of this research of significantly improving the performance of the EDM process by reducing the electro-erosion wear of the electrode material. The average grain size after thermoplastic processing optimisation was *d*_2_ = 320 nm.

A significant increase in strength relative to the material in the initial state equal to *YS* = 630 MPa (an increase of 56%) and a *UTS* = 646 MPa (an increase of 72%) was observed. At the same time, a slight decrease in electrical conductivity was observed to 78% *IACS*, a decrease of 3% relative to the material in the initial state. EDM wear tests conducted using two machining variants, i.e., rough and finish machining, demonstrated the significantly higher efficiency of the proposed solution in finishing machining. For this type of machining, an almost 50% lower electrode wear was observed after the HE process compared to the commercial material. In comparison, in rough machining, the value of electrode wear after HE was comparable to that of a conventional electrode. This effect was attributed to the phenomenon of degradation of the microstructure refinement in the HE process as a result of the high processing parameters generating high temperatures in the erosion zone, which does not occur in finish machining.

## Figures and Tables

**Table 1:** Chemical composition (wt.%) of the CuCrZr alloy.

Cr	Zr	Fe	Si	Cu
0.5–1.2	0.03–0.3	Max 0.08	Max 0.1	balance

**Table 2:** Properties of the CuCrZr copper alloy in the initial state and after solution treatment.

Material	Ultimate Tensile Strength, *UTS* (MPa)	Yield Strength, *YS* (MPa)	Elongation, *A* (%)	Hardness, *HV*_0.2_	Electrical Conductivity, *IACS* (%)
**CuCrZr in initial state**	413	366	19	153	84.7
**CuCrZr** **1000 °C/1 h**	220	90	45	101	32.3

**Table 3:** EDM machining parameters in wear tests.

Type of Machining	Working Amperage *I_r_* (A)	Pulse Time *t* (ms)	Depth of Machining *H* (mm)
**Rough**	12	9	20
**Finish**	3	4	20

**Table 4:** Basic parameters of the hydrostatic extrusion process of the CuCrZr copper alloy.

Specimen		Initial Diameter, *d*_0_ (mm)	Product Diameter, *d_f_* (mm)	True Strain, *ɛ* = ln*R* ^(a)^	Cumulative True Strain, *ɛ_cum_*	Adiabatic Temperature, *T* (°C)	*T/T_m_* ^(b)^	Hydrostatic Extrusion Pressure, *p_HE_* (MPa)
**1xHE**	**1CCZT**	69.44	39.85	1.11	1.11	127	0.29	480
	**2CCZT**	69.44	29.76	1.69	1.69	188	0.35	709
	**3CCZT**	69.44	19.76	2.51	2.51	285	0.41	1075
**5xHE**	**1CCZT**	69.44	39.85	1.11	1.11	119	0.29	480
	**1CCZT-2**	39.85	29.86	0.58	1.69	107	0.28	404
	**1CCZT-3**	29.86	19.89	0.81	2.50	144	0.30	542
	**1CCZT-4**	19.89	15.00	0.56	3.07	127	0.29	479
	**1CCZT-5**	15.00	9.98	0.81	3.88	169	0.32	638

^(a)^ *R*—reduction ratio = initial to final cross-section. ^(b)^ *T_m_*—melting point = 1084 °C.

**Table 5:** Mechanical properties and electrical conductivity of CuCrZr, processed as described in the present study, compared with the properties of this material treated with other techniques reported in the literature (ordered according to *UTS*).

Processing	Sample Size (mm)	True Strain, ɛ	Grain Size, d_2_ (nm)	UTS (MPa)	YS (MPa)	IACS (%)	Ref.
**ST + LNT-DPD ^(1)^ + aging 400 °C**	23 × 11 ^(3)^	2	-	832	-	71.2	[15]
**Annealing + LNT-DPD**	30 × 2.3 ^(3)^	2	-	700	-	78.5	[13]
**ST + HE × 5 + aging 420 °C**	10	3.89	210	679	676	71.8	This work
**ST + ECAP × 8 + aging 460 °C**	11.5 × 11.5	8	256	676	-	73	[47]
**ST + HE × 5 + aging 450 °C**	10	3.89	-	668	657	77.5	This work
**ST + HE × 5 + aging 480 °C**	10	3.89	320	646	629	78.0	This work
**ST + HE + aging 480 °C**	16	2.28	200	630	585	79.0	[34]
**ST + ECAP × 2 + HE + aging 480 °C**	16	3.6	180	625	610	78.0	[34]
**ST + HE × 5 + aging 510 °C**	10	3.89	425	615	590	80.3	This work
**Annealing + ST +cold rolled + aging 400 °C**	0.4 ^(2)^	98%	152	592	559	86.8	[48]
**ST + aging 450 °C +ECAP × 8 (200 °C)**	14 × 14	9.2	700	550	535	68.8	[16]
**ST + cold drawing + aging 450 °C**	10	-	-	550	510	78.7	[12]

^(1)^ dynamic plastic deformation (DPD) at liquid nitrogen temperature, ^(2)^ final plate thickness, ^(3)^ disk diameter × thickness.

## Data Availability

Experimental methods and results are available from the authors.
